# Effects of PM_2.5_ on Third Grade Students’ Proficiency in Math and English Language Arts

**DOI:** 10.3390/ijerph17186931

**Published:** 2020-09-22

**Authors:** Casey Mullen, Sara E. Grineski, Timothy W. Collins, Daniel L. Mendoza

**Affiliations:** 1 Department of Sociology, University of Utah, 480 S 1530 E. Rm 0301, Salt Lake City, UT 84112, USA; casey.mullen@utah.edu; 2 Department of Sociology/Environmental and Sustainability Studies, University of Utah, 480 S 1530 E. Room 0301, Salt Lake City, UT 84112, USA; 3 Department of Geography/Environmental and Sustainability Studies, University of Utah, 260 Central Campus Dr #4625, Salt Lake City, UT 84112, USA; tim.collins@geog.utah.edu; 4 Department of Atmospheric Sciences/City & Metropolitan Planning, University of Utah, 135 S 1460 E. Room 819, Salt Lake City, UT 84112, USA; daniel.mendoza@utah.edu

**Keywords:** air pollution, PM_2.5_, environmental justice, primary schools, academic proficiency, Salt Lake County, Utah

## Abstract

Fine particulate air pollution is harmful to children in myriad ways. While evidence is mounting that chronic exposures are associated with reduced academic proficiency, no research has examined the frequency of peak exposures. It is also unknown if pollution exposures influence academic proficiency to the same degree in all schools or if the level of children’s social disadvantage in schools modifies the effects, such that some schools’ academic proficiency levels are more sensitive to exposures. We address these gaps by examining the percentage of third grade students who tested below the grade level in math and English language arts (ELA) in Salt Lake County, Utah primary schools (*n* = 156), where fine particulate pollution is a serious health threat. More frequent peak exposures were associated with reduced math and ELA proficiency, as was greater school disadvantage. High frequency peak exposures were more strongly linked to lower math proficiency in more advantaged schools. Findings highlight the need for policies to reduce the number of days with peak air pollution.

## 1. Introduction

Emerging evidence suggests that air pollution negatively affects children’s cognitive development [1,2,3,4,5,6], and academic performance in primary school [7,8,9,10]. The extensive environmental justice (EJ) literature has documented that all children are not equally exposed. EJ studies focused on US schools have determined that schools with higher proportions of racial/ethnic minorities [6,11,12,13,14,15,16,17] and low-income students [11,13,14,16,18,19] experience worse air pollution than schools that serve predominately White and affluent students.

Previous studies linking air pollution and school-level academic proficiency have focused on chronic measures of air pollution, neglecting the frequency of peak exposures, and they consider air pollution exposures and student demographic characteristics to be independent correlates of academic proficiency, ignoring the potential for synergy between the two. We address those gaps with a focus on primary schools in Salt Lake County (Utah), a place with acute episodes of air pollution by particulate matter ≤2.5 microns in diameter (i.e., PM_2.5_). We use school-level data to address three aims: (1) to examine how social disadvantage is related to chronic exposures and the frequency of peak exposures to PM_2.5_; (2) to test how chronic PM_2.5_ exposure and the frequency of peak PM_2.5_ exposures impact student academic proficiency in math as well as English language arts (ELA); and (3) to test for synergies between PM_2.5_ exposures and social disadvantage in predicting academic proficiency.

### 1.1. Impacts of Air Pollution on Children’s Cognition and School Performance

Air pollution exposure is unhealthy for humans, especially children. Children are more sensitive to air pollution exposure than adults because they are still developing and their smaller body size increases their breathing rate [5]. Increased exposure to traffic-related air pollution is associated with respiratory problems [6], such as asthma and wheezing [20]. Children can also suffer from neurological effects due to air pollution [2,4,5]. This results in DNA damage [21], increased risk of autism [22], and neurobehavioral dysfunction and developmental delays [1]. Research relying on autopsies of Mexico City children who were chronically exposed to high levels of air pollution found evidence of Alzheimer’s disease-associated pathology [2,4]. Chronic exposure to air pollution is associated with white matter hyperintensities, i.e., myelin damage to nerve cells from reduced blood flow, which impairs nerve cell communication through synapses [5].

Studies at the school level have associated air pollution exposure with group-level differences in academic proficiency. In the Los Angeles Unified School District of California, closer proximity to point-source industrial facilities and higher concentrations of ambient air toxics known to harm the respiratory system in each schools’ host census tract were significantly associated with schools having lower academic performance index (API) scores [23]. The same pattern was found statewide [16]. Higher PM_2.5_ concentration (measured as a three-year average, 2009–2011) has also been associated with lower 2010 API scores at schools in the Sacramento area located near roadways [14]. Michigan public schools exposed to industrial toxics in 2006 had the highest proportion of students who failed to meet state testing standards in ELA and math in 2007, adjusting for other factors [24]. A more recent study in Michigan also found a similar pattern [6]. In addition to this school-level literature, a handful of studies have examined individual children’s academic performance in relation to air pollution exposures. Annual air pollution exposures among primary school children have been linked to lower grade point averages [25], and lower reading [7,9], science [7] and math standardized test scores [7,8].

Those school- and individual-level studies of academic performance have exclusively examined measures of chronic air pollution, like annual averages of air pollutant concentrations [24] or proximity measures, such as the distance to nearest roadway [6]. Most studies have analyzed only one chronic measure of pollution [26], neglecting multiple exposures. While chronic exposure to air pollution is health harming, many studies document how short-term exposures (e.g., daily increases) are linked to physical health effects in children [27,28]. Studies are also starting to document short-term cognitive effects. For example, one study found that commuting outdoors near a major roadway predicted same-day declines in memory and attention span for adults [29]. Children’s cognition is also affected. Sunyer et al. [30] found an association between the daily increases in traffic-related air pollution and reduced attention-spans in primary school children. In terms of academic performance, the poor air quality on the day of a standardized test measurably reduced Israeli high school students’ test scores [31].

Thus, it is plausible that more frequent peak air pollution exposures (e.g., when daily PM_2.5_ rises well above the mean) are associated with declines in academic performance, but this has never been tested. A study that compared the relative strength of a variety of total suspended particulate (TSP) air pollution metrics on respiratory health effects found that the high exceedance frequency metric (i.e., number of hours above 200 pg/m^3^) had a larger effect than the 24 h mean concentration of TSP [32]. In a study of cancer in women, researchers observed that the risk of cancerous tumors rose with increasing exceedance frequencies (e.g., number of hours above federal air quality standards) for total suspended particulates [33]. To the best of our knowledge, exceedance frequency metrics have never been examined in studies of school-level academic proficiency.

### 1.2. Children’s Differential Sensitivity to Air Pollution

Some studies have revealed that certain social groups are more sensitive than other groups to negative health effects arising from pollution exposures. Counterintuitively, some researchers have discovered that, in spite of their lower exposures, residents of socially advantaged neighborhoods (e.g., White and affluent) are more sensitive to the negative health effects of air pollution than are residents in socially disadvantaged neighborhoods [34,35,36,37]. Higher levels of environmental pollution exhibited stronger associations with mortality in more affluent communities than less affluent communities in the UK [36] and in Sao Paolo, Brazil [37]. Gouveia and Fletcher [37] hypothesized that their findings related to how residents in more affluent districts “were more ‘protected’ from other important causes of death” (p. 754). In El Paso, Texas, wheezing symptoms among primary school children were more closely connected to air pollution when the children were socially advantaged, non-Hispanic White, and living in areas with lower levels of air pollution [34]. Grineski et al. [34] hypothesized that their findings were attributable to the fact that “the relationship between air pollution and health is not complicated by social deprivation and the challenges of poverty” (p. 38). In other words, if all members of the population had equal access to health-promoting resources (e.g., medical care, healthy homes, a basic living wage), then we would expect outdoor environmental exposures to play a more important role in explaining geographic disparities in health then they would in socially unequal communities, wherein social determinants largely explain health disparities [38].

While those studies have paradoxically found that residents in less exposed/wealthier/Whiter areas are more sensitive to the health effects of air pollution than residents in more highly exposed/poorer/minority areas, others have found the opposite. Some have found that racial/ethnic minority and low-income people are more sensitive to the health effects of air pollution than are White and more affluent people [39,40,41,42,43]. For example, in Phoenix, children without insurance (i.e., low-income children) had a 1.4 times higher risk of asthma admissions than those with private insurance (i.e., higher income children) when levels of NO_2_ were high [44].

A few studies on cognitive outcomes demonstrate similar findings. Poor air quality was associated with greater impacts on adult cognitive ability among persons with low socioeconomic status (SES) [45] and older adults living in poorly maintained, neglected neighborhoods [46]. Low socioeconomic status compounded risks of prenatal polycyclic aromatic hydrocarbon exposure on attention-deficit/hyperactive disorder symptoms [47]. However, the question as to how sociodemographic factors modify the associations between air pollution exposures and academic endpoints remains unanswered. We take a first step toward filling this important knowledge gap by examining the relationships for two measures of air pollution—chronic exposure and the frequency of peak exposures to PM_2.5_—with school-level academic proficiency.

Our study answers three questions using school-level data on third grade students from all Salt Lake County, Utah public elementary schools. We first examined patterns of distributive environmental injustice with respect to chronic exposures and the frequency of peak exposures to PM_2.5_, which is a well-known cause of neurological health effects [48]. We used a measure of school disadvantage, which combines racial/ethnic minority composition with economic deprivation, to answer our first question: is school disadvantage associated with chronic PM_2.5_ exposures and the frequency of peak PM_2.5_ exposures? While previous studies of school-level exposures and academic proficiency have focused on chronic exposures, we also examined the effects of more frequent peak exposures to answer our second question: are chronic PM_2.5_ exposures and/or the frequency of peak PM_2.5_ exposures related to lower proficiency in math and English and language arts (ELA), net of school disadvantage? Third, we examined the combined (interactive) effects of school disadvantage and air pollution exposures on proficiency in math and ELA. Time-series air pollution epidemiology studies have examined how social factors modify the effects of pollution on health [39]. However, school-based studies on air pollution and academic proficiency have not, which highlights the relevance of our third question: does school disadvantage modify the effects of chronic PM_2.5_ exposures and/or the frequency of peak PM_2.5_ exposures on proficiency in math and ELA?

The project qualifies as exempt human subjects research under category 4 as we use a secondary data set, which is publicly available, and the individual subjects cannot be identified directly or through linked identifiers because their information is aggregated at the school-level.

## 2. Materials and Methods

### 2.1. Case Study Area

We conducted our study in Salt Lake County, the most populous county in Utah, with a 2017 population of 1,135,649. Salt Lake County is also home to a relatively diverse population. Data from the American Community Survey (ACS) from 2017 indicates that although the population is predominantly (70.93%) non-Hispanic/Latino White, it also includes more than 330,131 (29.06%) racial/ethnic minority residents, with Hispanic/Latino as the largest minority group at 18.28% of the population. Two distributive environmental injustice studies on unequal exposures to air pollution have been conducted in the Salt Lake area to date, and they have revealed strong patterns of injustice with respect to race/ethnicity [11,12]. One study relied on chronic, averaged air pollution measures [12], and the other examined three different two-day air pollution scenarios [11]. A local study on Salt Lake schools found associations between PM_2.5_ concentrations and daily rates of school absenteeism [49].

Episodic PM_2.5_ air pollution is a serious problem in Salt Lake County. The growing population, car-dependent culture, and its geographic location contribute to its air pollution problems. The unique geography of the Salt Lake Valley leads to frequent temperature inversions that inhibit the dispersion of ground-level emissions, particularly in the winter months. The region has been in federal non-attainment for PM_2.5_ from 2009 to 2020 [50]. According to the most recent American Lung Association’s “State of the Air” report, which examines both annual averages and 24 h, daily spikes throughout the year, Salt Lake City (within Salt Lake County) was among the top 10 most polluted American cities in regard to short-term spikes of unhealthy particle pollution during the period 2016–2018 [51]. The negative health impacts of pollution in Salt Lake County have been well documented [52,53,54,55,56,57].

### 2.2. Unit of Analysis

**Figure 1:**
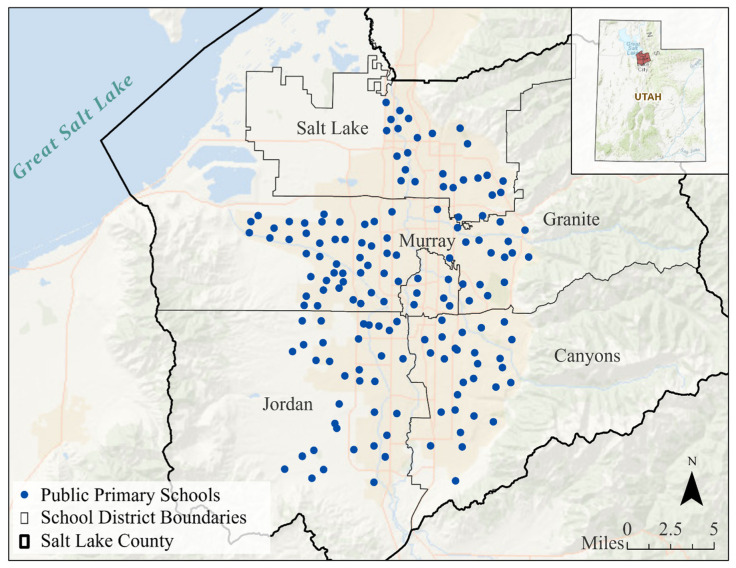
Salt Lake County case study area map showing the locations of Salt Lake County public primary schools (*n* = 156) and school districts (*n* = 5).

The unit of analysis for our study was Salt Lake County public primary schools that offered third grade. The schools are located in five different school districts and are depicted in Figure 1. While there are 162 such schools, 156 were included in this analysis as they reported data to the Utah Education Association about their students’ Student Assessment of Growth and Excellence (SAGE) test scores for both math and ELA. School-based exposures are important to assess because students are located in a singular location while they are at school [6]. Our focus on third graders is warranted since children’s cognitive functioning matures between the ages of 6 and 10 years [58,59]. Third graders are 8 and 9 years old on average and thus included in that critical developmental window.

### 2.3. Air Pollution Metrics

For our first research question, we used two PM_2.5_ metrics as dependent variables_._ We present descriptive statistics for all the variables used in the analysis in Table 1. We derived the two PM_2.5_ metrics from the 2016 US Environmental Protection Agency Downscaler PM_2.5_ daily census tract-level data. PM_2.5_ concentrations were estimated using a Bayesian space-time downscaling fusion model [60]. The Downscaler method integrates data from a gridded atmospheric model (i.e., Community Multi-Scale Air Quality Model or CMAQ) and point air pollution measurements from the National Air Monitoring Stations/State and Local Air Monitoring Stations (NAMS/SLAMS). CMAQ estimates gridded averages with no missing values but is subject to calibration error; that error is accounted for through fusion modeling with monitoring data from NAMS/SLAMS that provide direct, accurate measurements of air pollutants. The Downscaler daily PM_2.5_ concentrations correspond to 2010 census tract geographic identifiers [61], and have increasingly been employed in air pollution epidemiology studies [62,63,64].

**Figure 2:**
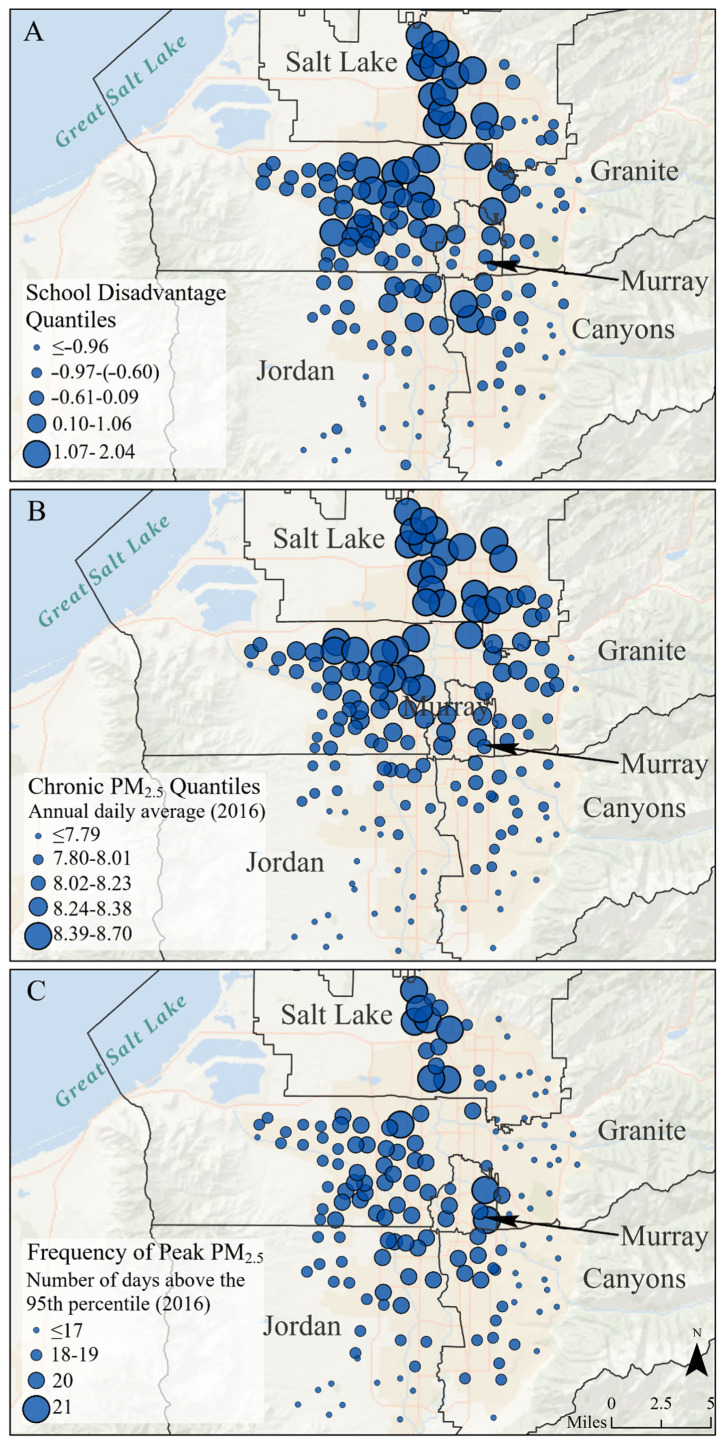
Three maps depicting school disadvantage (**A**), chronic PM_2.5_ pollution (**B**), and the frequency of peak PM_2.5_ pollution (**C**) at Salt Lake County schools (*n* = 156). Note: School disadvantage is presented in units of standard deviation. Chronic PM_2.5_ is presented in units of µg/m^3^. Peak PM_2.5_ represents the number of days that the school tract’s PM_2.5_ level was over the 95th percentile of the daily mean of all tract days in Salt Lake County (i.e., 22.80 µg/m^3^) and is presented in units of days.

To create both dependent variables, we first downloaded PM_2.5_ concentration estimates for all Salt Lake County census tracts for each day of the year. Then, we located each school in its host census tract and assigned the school the PM_2.5_ values from the host tract. The chronic air pollution metric is the daily average of the modeled PM_2.5_ concentrations (μg/m^3^) in each school’s tract for all days in 2016. On average, schools had chronic PM_2.5_ levels of just over 8 micrograms per cubic meter (range: 6.66–8.70 μg/m^3^). The frequency of peak exposure metric is the number of days (in 2016) that the school tract’s PM_2.5_ concentration was over the 95th percentile of the daily mean of all tract days in the county (i.e., 22.80 μg/m^3^). The average school had 18.50 days above the 95th percentile (range: 11.00–21.00 μg/m^3^). Both variables are mapped in Figure 2. We use these two pollution metrics as dependent variables in research question 1, and as independent variables to answer research questions 2 and 3.

### 2.4. Academic Proficiency Variables

We used two academic proficiency variables as dependent variables for the second and third research questions. These variables are the percentages of third-grade students at each school that tested below grade-level proficiency for math and for ELA at the end of the 2016–2017 school year. Below proficiency is determined based on the student performance on the Student Assessment of Growth and Excellence (SAGE) computer-adaptive standardized tests, which were used by the Utah Education Association (UEA) to assess core competencies. UEA reports SAGE student test results as scale scores (measures of what the students know) as well as proficiency scores. We used proficiency scores because they interpret the scale scores in relation to grade-level expectations. In Salt Lake County (2017), the average school had 31.79% of students below proficiency in math (range 2.53% to 74.67%) and 30.99% of students below proficiency in ELA (range of 2.53%–81.25%).

### 2.5. Sociodemographic Variables

We acquired sociodemographic data pertaining to each school from the National Center for Education Statistics’ (NCES) ElSi tool. This tool provides users with access to the US Department of Education’s primary database on public elementary and secondary schools in the United States through the Common Core of Data (CCD). We downloaded data on each school’s Title I school status (whether a school was eligible for Title I or not); the percentage of Hispanic students; the percentage of non-Hispanic minority students who were Black, Asian/Pacific Islander, or Native American/Alaska Native; and the percentage of students on free and/or reduce-priced meals. Previous EJ studies focusing on air pollution and schools have examined these variables [11,13,16,17,19,24,65].

While we ideally would have examined those four variables separately, they exhibited multicollinearly due to high correlations (e.g., the correlation coefficient between percent Hispanic and percent on free and reduced-price meals was 0.938). We created a school disadvantage factor that combined the four variables. The factor had a Cronbach’s alpha of 0.724, indicating adequate reliability. We used school disadvantage as an independent variable in all analyses. It is mapped in Figure 2.

### 2.6. School Characteristics

In the analyses for each of the three research questions, we controlled for school enrollment and school district. We obtained school enrollment (2016–2017 school year) from NCES and included a standardized version of this variable in the models. The schools under analysis are located in all five Salt Lake County school districts, which we included categorically in the analysis. These are Canyons, Granite, Jordan, Murray, and Salt Lake City (with Salt Lake City as the reference category).

### 2.7. Generalized Estimating Equations

Our analyses used generalized estimating equations (GEEs), which are well suited for clustered and non-normally distributed data, as they provide a better fitting model than regular ordinary least squares (OLS) regression [66,67]. Other EJ studies on air pollution have utilized GEEs in their analyses [11,13,68].

GEEs depend on the definition of data clusters [66], with the assumption that observations in a cluster are related and observations from other clusters are independent from one another [66]. We defined clusters in our GEEs based on school district precincts and the median age of housing stock corresponding with each schools’ host census tract. We used precincts, which are sub-districts mapping to a school board representation within each district. We used this as a clustering definition instead of school districts, since the school districts in the study were dramatically different in size (range: six to 62 schools), resulting in a small number of uneven clusters. Within the five districts there were 28 precincts, with the number of primary schools in each ranging from three to 12. We also used a median age of housing stock derived from the 2017 five year American Community Survey estimates as a clustering variable to account for the geographic context in which each school was located. We assigned each school the median age of housing stock of its host tract, based on eight median years of construction categories (i.e., pre-1940, 1940–1949, 1950–1959, 1960–1969, 1970–1979, 1980–1989, 1990–1999, 2000+). This clustering variable is commonly used in urban EJ studies. Homes are often spatially clustered by the period of construction and EJ research has shown that this is a relevant variable that corresponds to the exposure to environmental harm [12,68,69,70].

GEEs require the specification of a working correlation matrix, a distribution, a link function [66,67], and a quasi-likelihood under independence model criterion (QIC) goodness-of-fit coefficient is used to determine the best fitting combination of the specifications for a given GEE model [66]. We tested the unstructured, exchangeable, and independent working correlation matrices with identity and logarithmic link functions for all models. For models predicting chronic PM_2.5_, the percentage below proficient in math and the percentage below proficient in ELA, we tested normal, gamma, and inverse Gaussian distributions. We tested negative binomial and Poisson distributions for the model predicting the frequency of peak PM_2.5_.

To answer research question 1, we specified two models, one for each dependent variable (i.e., chronic (Model 1) exposures and the frequency of peak exposures (Model 2)) using school disadvantage and school characteristics. These models required different specifications. Model 1 utilized an exchangeable working correlation matrix with an inverse Gaussian distribution and an identity link function. This working correlation matrix specification has been used in similar EJ studies [13,68]. Model 2 used an unstructured working correlation matrix with a negative binomial distribution and an identity link function. The negative binomial distribution was appropriate because the dependent variable represents a count, in this case, the number of days a school’s tract 24 h PM_2.5_ concentration was above the 95th percentile [66]. The general form of quasi-likelihood estimating equations is given by

(1) ∑i(∂μi∂β)′v(μi)−1[yi−μi(β)]=0

where $\mu_{i}=g^{-1}(X^{\prime }\beta )$ is the link function with *g* = *log* or *g = identity* (depending on the model), the distribution is $y_{i}$, and the GEE estimator $\left(\hat{\beta }\right)$ is the solution to these equations. The resulting robust covariance of the GEE is given by

(2) $$V_{G,n}=n{\left[\underset{i}{\sum }D_{i}^{\prime }V_{i}^{-1}D_{i}\right]}^{-1}\left[\underset{i}{\sum }D_{i}^{\prime }V_{i}^{-1}{\mathrm{cov}(Y}_{i}{)V}_{i}^{-1}D_{i}\right]{\left[\underset{i}{\sum }D_{i}^{\prime }V_{i}^{-1}D_{i}\right]}^{-1}$$

and is assumed to be compound symmetric (exchangeable).

For research question 2, we used a stepwise modeling approach. We predicted math proficiency using chronic pollution and school characteristics (Model 3) and then we substituted out chronic pollution for the frequency of peak exposures (Model 4). The chronic and peak air pollution independent variables could not be included in the same model due to multicollinearity (they were correlated with each other at 0.550, *p* < 0.001). The setup of Model 5 and Model 6 was the same as 3 and 4, with the addition of the school disadvantage variable in each model to examine if air pollution negatively impacted the academic proficiency net of school social disadvantage. We repeated the same process for ELA (Models 8–11). For those models, an unstructured working correlation matrix with a normal distribution with an identity link function fit best. A normal distribution was used in association with normally distributed dependent variables (i.e., math and ELA academic proficiency) and the identity link function allows for the dependent variable to be predicted directly [66].

To answer research question 3, we added interaction terms to Model 6 and Model 11 resulting in two additional models, one for each dependent variable (math (Model 7) and ELA (Model 12)). We interacted school disadvantage with peak PM_2.5_ to examine if school disadvantage modified the effects of peak PM_2.5_ on academic proficiency. We did not include an interaction with the chronic PM_2.5_ independent variable as it was not significant in models 5 or 10.

To interpret the interaction effects, we used simple slopes tests. Simple slopes are used to predict the relationship between the X (the independent variable: frequency of peak PM_2.5_) and Y (the dependent variables: low math and ELA proficiency) at different levels of Z (the moderator variable: school disadvantage) [71]. Here, we examined if associations between the peak PM_2.5_ and the percent below proficient are significant at low disadvantage (1 standard deviation below the mean), mean disadvantage, and high disadvantage (1 standard deviation above the mean). We used Dawson’s tool for calculating simple slopes [71]. We input values pertaining to the variance of the independent variable (frequency of peak PM_2.5_) and the interaction effect (frequency of peak PM_2.5_ X school disadvantage) coefficients, the covariance value of the independent variable and the interaction effect coefficients, and the sample size (*n* = 156 schools) along with the number of control variables (*n* = 5).

## 3. Results

### 3.1. Associations between School Disadvantage and PM_2.5_ Exposures at School

Table 2 shows that increases in school disadvantage were significantly and positively related to both higher chronic PM_2.5_ exposures (*p* < 0.001) and greater frequency of peak PM_2.5_ exposures (*p* < 0.001). Note that the effect sizes cannot be compared directly due to different units for the dependent variables and model specifications between the two models. However, by dividing the coefficient by the range (and multiplying by 100), we can obtain the percent increase in PM_2.5_ associated with a standard deviation increase in school disadvantage, which is comparable between the two metrics. As such, 0.14 unit change in chronic PM_2.5_ represents a 6% increase in PM_2.5_, based on the range (2.04 μg/m^3^) and an additional 1.28 days above the 95th percentile for PM_2.5_ represents a 13% increase, based on the range (10 days). This suggests that the peak PM_2.5_ was more strongly associated with school disadvantage than was chronic PM_2.5_.

### 3.2. Associations between Chronic PM_2.5_ Exposure and the Frequency of Peak PM_2.5_ Exposures on Academic Proficiency

Table 3 reports all the results for math while Table 4 includes all the ELA results. While higher levels of chronic air pollution were associated with higher percentages of children who tested below proficient in math (*p* < 0.001), the effect became statistically insignificant with the addition of school disadvantage (Model 3). The effect size for chronic pollution became 78% smaller with the addition of school disadvantage, which was positive and significant (*p* < 0.001) (Model 5). We obtained the 78% figure by subtracting from one the coefficient from Model 5 (6.49) divided by the coefficient from Model 3 (28.70) and then multiplying that figure by 100. In terms of the association between school disadvantage and math proficiency, a one standard deviation increase was associated with 11.7% more children being below proficient, net of chronic air pollution and school characteristics (Model 5).

In terms of the frequency of peak pollution, the variable was significant (*p* < 0.001) without adjusting for school disadvantage (Model 4) and it retained statistical significance (*p* = 0.012) with the addition of school disadvantage (Model 6), which was also positive and significant (*p* < 0.001). The addition of school disadvantage reduced the effect size of peak pollution by 64%. Nonetheless, one additional day of peak pollution resulted in an additional 1.5% of children in school being below proficient in math (*p* = 0.012), net of school disadvantage and school characteristics (Model 6).

In order to compare the power of the two pollution metrics, we compared the ratios of the parameter estimate to the estimated standard errors of the coefficient [32]. The ratios were 1.69 (Model 5) for chronic exposures and 2.52 (Model 6) for peak exposures. As 2.52 is larger than 1.69, this comparison suggests that the frequency of peak exposures was associated more strongly with math proficiency than was chronic exposures. Both ratios were smaller than the ratio for school disadvantage, which was >9 in both of those models. This suggests that school disadvantage was more strongly related to math proficiency than was either pollution measure.

We found a similar pattern when predicting low proficiency in ELA (Table 4). Chronic pollution was significant (*p* < 0.001) and positive (Model 8) and became insignificant after the addition of school disadvantage (which reduced the effect size by 20%) (Model 10). In that model, a one standard deviation increase in school disadvantage was associated with 11.5% more children being below proficient in ELA (*p* < 0.001), net of chronic air pollution and school characteristics. As was the case with math, the frequency of peak pollution was significant (*p* < 0.001, Model 9) and it retained significance even after the addition of school disadvantage (*p* = 0.015, Model 11). Comparing the size of the coefficients between models 9 and 11, we can see that the effect of peak pollution on ELA was 54% smaller after the addition of school disadvantage. Even after that reduction, one additional day of peak pollution resulted in 1.8% more children being below proficient in ELA (*p* = 0.015), adjusting for the effects of school disadvantage and school characteristics (Model 11).

In terms of the effect size comparisons between the two metrics, the ratio of the parameter estimate to the standard error showed that the frequency of peak exposures had a larger effect (2.4, Model 11) than chronic exposures (1.03, Model 10). As with math, both effect sizes were smaller than school disadvantage (>7 in both models).

### 3.3. Interactions between PM_2.5_ Exposures and School Disadvantage in Predicting Academic Proficiency

Model 7 (Table 3) reports results from the GEE which included the interaction of frequency of peak PM_2.5_ and school disadvantage (*p* = 0.007) when predicting the percentage of children with math proficiency below grade level. We calculated simple slopes to examine this significant effect. When school disadvantage was high (i.e., 1 standard deviation above the mean), the slope was slightly negative (−1.10) and not significant (*p* = 0.301). This meant that the frequency of peak PM_2.5_ and low math proficiency were not significantly related at highly disadvantaged schools. When school disadvantage was average, the slope was slightly positive (1.07), but not significant (*p* = 0.286). When disadvantage was one standard deviation below the mean, which reflects low disadvantage, the relationship between higher frequency peak PM_2.5_ and lower math proficiency was positive (2.38) and significant (*p* < 0.001).

Model 12 (Table 4) reports results from the GEE including the interaction of peak PM_2.5_ frequency and school disadvantage when predicting ELA. The interaction was not significant although the directionality of the coefficients was similar to the math model, suggesting that associations between a higher frequency peak pollution and higher percentages of children with low ELA proficiency are stronger in more advantaged schools, but not significantly stronger. We did not run interactions between chronic pollution and school disadvantage for math or ELA since the chronic pollution variable was not significant in the full model (Models 5 and 10).

## 4. Discussion

In answer to our first question, we found that greater school disadvantage was significantly predictive of increased chronic PM_2.5_ exposure as well as the frequency of peak PM_2.5_ exposures at schools. School disadvantage was more strongly related to peak exposures than chronic exposures (e.g., the ratio of the coefficient to the standard error was 8.10 in the model predicting the daily annual average and 11.10 in the model predicting the number of peak days). Our findings associating racial/ethnic minority composition and economic deprivation (combined into one index) with the chronic PM_2.5_ metric (i.e., annual daily average) aligns with other studies in Michigan [6,24], and California [14,16,23]. While our peak PM_2.5_ findings align with our findings from the model predicting chronic PM_2.5_, they illustrate another dimension of environmental injustice that is less often investigated. How those peak exposures might translate into negative effects for children is still an open question. In terms of how they affect academic proficiency at the school level, we address that with our second question.

In answer to our second question, chronic annual average exposure was positively related to the percentage of students with low proficiency in math and ELA initially, but those significant effects were explained away by the inclusion of social disadvantage. Unlike chronic exposures, the frequency of peak exposures was positively associated with the percentage of students with low proficiency in math and ELA, even after accounting for school disadvantage. The effect size of the acute exposure coefficient was similar between the math and ELA models. This has been found in other studies [7,24], although some have found that math [10] or verbal abilities [45] are more affected by air pollution.

Why peak exposures were more closely related to low academic proficiency than chronic exposures is an open question. Certainly, in Salt Lake County, those two metrics are related to each other (Pearson’s correlation = 0.550). It is possible that repeated peak exposures have more severe impacts on the brain than chronic exposures. As air pollutants enter the body, they induce neuroinflammation as a result of activated microglia, the immune cells of the brain that regulate neuroinflammation [72]. Neuroinflammation contributes to cell loss within the central nervous system, which is believed to be linked to cognitive deficits [5]. It may be that neuroinflammation triggered by frequent peak exposures is more damaging to children’s brains than the effects caused by a lower concentration of daily exposures. Relatedly, research on peak exposures in cars has shown that in-vehicle PM_2.5_ contains a high level of chemicals that causes oxidative stress. The body responds similarly to these chemicals and the PM_2.5_ to cope with the reactive oxygen species contained in both. The dual exposure leads the body to overreact, which may be destructive to DNA [73]. A similar overreaction could be behind the damaging effects of peak exposures at school. However, these hypotheses are tentative.

These results contribute to a growing body of literature suggesting that PM_2.5_ standards are not low enough [74,75,76]. In this case, we defined peak exposures as the 95th percentile, which was just under 23.00 μm^3^; the federal 24 h standard is 35.00 μm^3^. While not focused on lowering the PM_2.5_ standard, the State of Utah has taken steps to reduce peak exposures to PM_2.5_. Recognizing that mass transit is key to reducing traffic pollution, the state passed House Bill 0353 in 2019, which provides free public transit fees on “red air” days to encourage ridership and reduce the use of personal vehicles [77]. The State is also trying to better quantify the environmental impacts of new developments and address those impacts on proximate communities, as evidenced by the passage of Sen. Escamilla’s Senate Bill 0112 in 2020 [78]. We concur with the assertion of Pastor et al. [16] that, “in some sense, schools are a barometer for society as a whole: Improving air quality with kids in mind can improve air quality for everyone” (p. 356).

School disadvantage was a strong and robust predictor of having lower proficiency in math and ELA, controlling for the other variables in the model (including pollution). Our results align with extant knowledge about how social factors are critical in predicting academic proficiency. The problem of poverty (measured by the proportion of students on free and reduced-price meals and Title 1 status in our index) often shares a dynamic relationship with poor health outcomes [23]. Poor students may lack access to proper nutrition, healthcare and medication, which all may negatively impact academic outcomes [16,17,23]. Poor parents often have lower educational attainment than more affluent parents, and parental educational attainment is an important social factor shaping academic proficiency [16,17,23]. Racial/ethnic minority students may have an unequal educational experience compared to White students as a result of differential treatment and stigmatization. For example, racially biased interactions with teachers and peers negatively impact racial/ethnic minority students’ academic experiences [79]. Differential teacher treatment is witnessed through teachers having higher expectations of White and Asian students and lower expectations of Black and Hispanic students as well as teachers providing more positive feedback to White students than racial/ethnic minority students [80]. Steele and Aronson [81] found that Black students were subject to a stereotype threat effect that caused them to underperform on tests in comparison to White students. An awareness of racial stereotypes about Black students’ intellectual ability and feelings of membership in a stigmatized group contributed to reduced test performance [81], and a recent study testing Black third graders had similar findings [82]. Racial/ethnic minority students are also sometimes immigrants or children of immigrants and English language learners. This is an added educational challenge [79], especially when standardized tests are administered in English [16,17,23].

Results addressing our second question, in terms of the insignificant finding for chronic pollution as well as the significant findings for peak exposures and school disadvantage, shed light on the social and environmental structure of academic disparities in this particular context and might be relevant for the design of interventions. In order to improve math and ELA proficiency, it is imperative to reduce barriers that accompany low incomes and racial/ethnic minority status. This insignificant finding does not necessarily indicate that chronic environmental exposures have no actual influence on academic proficiency. Several prior individual-level studies have shown that they do [7,8,9,10]. What our findings show is that social disadvantage fully encompasses the effect of chronic exposures on academic proficiency in Salt Lake County, but that is not the case with peak exposures. When accounting for environmental and social inequalities—rather than demonstrating that one factor is important and the others are not—results from such analyses highlight the multiple forms of jeopardy that affect children [83].

In answer to the third question, we found synergies between the peak PM_2.5_ exposures and school disadvantage for math, but not for ELA. Advantaged schools’ math proficiency was significantly and negatively impacted by higher frequency peak PM_2.5_ exposures, but the frequency of peak exposures did not have a significant impact on math proficiency in disadvantaged schools. While we found that more socially advantaged schools were relatively more affected by additional days of peak pollution, it is important to note that, in absolute, unadjusted terms, higher proportions of students were below proficient in socially disadvantaged schools. At socially disadvantaged schools, 45.56% and 45.82% tested below proficient in math and ELA, respectively, while those corresponding percentages were 15.40% and 13.78% at socially advantaged schools. It is also the case that advantaged schools (i.e., those ≤ one standard deviation below the mean) averaged 16 peak days in 2016 while the disadvantaged schools (i.e., those ≥ one standard deviation above the mean) averaged 20 days; this difference was statistically significant (*p* < 0.001) as per an independent samples t-test (table not shown).

This interaction effect suggests that the effect of peak PM_2.5_ exposures on decreased math proficiency in the base model was driven by the stronger association in more advantaged schools. This is presumably because in more disadvantaged schools there are more influential social determinants of low academic proficiency, closely related to economic deprivation and minority race/ethnicity, e.g., food and housing insecurity, reduced access to quality physical and mental health care, experiences with discrimination, and financial stress [79,84,85,86]. Those factors are unmeasured in our models, but they likely combine as a constellation of risks that influence children’s academic proficiency in disadvantaged schools to a greater degree than air pollution, even though PM_2.5_ exposures are higher. In contrast, in more socially advantaged schools, that constellation of risk factors is much less present, enabling variation in air pollution to exert a stronger association with academic proficiency.

Pearce et al.’s [36] findings suggest that environmental predictors mattered more in wealthy areas than in poor areas because health needs (e.g., access to health care, medications, housing, and nutrition) were already being met. Grineski et al. [83] posed the following hypotheses, after finding that social factors explained away the effect of air pollution on asthma hospitalization rates in El Paso Texas: “if all members of a population had equal access to needed health resources (e.g., medical care, healthy homes), outdoor environmental exposures would play a more important role in explaining geographic disparities in health than they do in socially unequal communities” (p. 43). In this case, it seems like our interaction effects findings for math provide some support for that hypothesis. It is worth noting that while the interaction between school disadvantage and peak pollution was not statistically significant in the ELA model, the directionality of the findings aligns with the math results.

Why the interaction was significant when predicting math but not ELA is unclear. A recent national Chinese study found that verbal abilities were more affected by air pollution exposure—measured as a city-level index of sulfur dioxide, nitrogen dioxide, and particulate matter equal to or smaller than 10.00 μm (PM_10_)—than were math abilities across two waves of data collection, i.e., 2010 and 2014, in an ‘all ages’ sample [45]. It is possible that ELA abilities are more directly affected by pollution such that school disadvantage is less of a modifier of that associations. However, other studies have found children’s math abilities to be more affected by PM_2.5_ [87]. Clearly, additional research on how pollution affects student performance in different subject matter areas is needed.

### Limitations and Future Directions

Our study has some limitations. We only examined outdoor air pollution at schools. However, it is established that personal exposure to air pollution is driven primarily by outdoor pollution levels [88], and that indoor and outdoor air pollution are highly correlated [89,90,91,92,93]. Future research should seek to incorporate indoor and outdoor measures of pollutants at schools.

There are also limitations with the SAGE testing data. Test dates are not available and so we are unsure exactly when the tests were conducted beyond the “end of the school year”. To provide an estimation of pollution exposures that might be affecting the children, we used the year before the test, which is imprecise yet captures the general levels of PM_2.5_ air pollution at each school. We were not able to include any measures of air pollution before 2016, even though it is well established that early exposures are harmful [28,94,95], as we were not able to track individual children as they progressed through primary school. Using panel data on individual children is an important next step to address many of these limitations.

## 5. Conclusions

The findings that we uncovered lend themselves to generally applicable practical applications to reduce academic and environmental disparities. Firstly, academic barriers that are present in low-income and minority schools may be reduced through educational policies that are based in equity as opposed to equality because equality emphasizes equal treatment while equity emphasizes the equality of outcomes [96]. We can also advance public health initiatives that target children’s safety from air pollution exposure. Implementing urban design that reduces air pollution from the perspective of the lived-experiences of children and their caretakers may lessen air pollution exposure. For example, the Gehl company specializes in designs such as Thrive Zones that work with urban planners to help incorporate cleaner air routes for children’s movement in communities [97]. Better ventilation and filtration systems can be installed in schools. States can enact regulations that prevent new schools from being built near known pollution sources as well as remediate exposures at schools already located near known pollution sources [16]. Additionally, adding vegetation barriers near schools in high-traffic zones can help to reduce near-road pollution exposure [98]. Regulations that limit bus and vehicle idling near schools as well as requiring cleaner burning fuels in busses can also reduce students’ exposures to fine particulates [16].

Increasing awareness of air pollution might be particularly important at more socially advantaged schools, given that there are fewer spikes in PM_2.5_ there, yet they play a role in children’s math proficiency. The population’s perception might be that PM_2.5_ is a more pressing problem in lower income/minority parts of Salt Lake County; and while that is true (as per Models 1 and 2), it does not mean that the cognitive capacities of students attending advantaged schools are unaffected. Indeed, there is mounting evidence that the negative effects of PM_2.5_ on the human body are present at even low levels, such that there may be no “safe” level of exposure to PM_2.5_ [74,75]. The dose–response curve may be steepest at the lowest levels of exposure [99]. Perhaps widespread recognition by socially advantaged populations of such risks will help catalyze environmental decision-making to protect all children from air pollution.

## Figures and Tables

**Table 1:** Descriptive statistics of analysis variables (*n* = 156 schools).

Variables	Yes (*N*, Number of Schools))	No (*N,* Number of Schools)	*N* (Number of Schools)	Minimum	Maximum	Mean	Standard Deviation
**Air Pollution Variables,**							
Chronic PM_2.5_^a^			156	6.66	8.70	8.08	0.38
Frequency of peak PM_2.5_^b^			156	11.00	21.00	18.50	1.80
**Academic Proficiency Variables**							
Percentage below proficiency in math (Spring 2017)			156	2.53	74.67	31.79	16.78
Percentage below proficiency in English and language art (Spring 2017)			156	2.53	81.25	30.99	17.61
**Sociodemographic Variables**							
School disadvantage factor (2016–2017)			156	−1.26	2.04	0.00	1.00
Percent free/reduced-price meals			156	8	99	46.83	27.95
Percent Hispanic			156	2	74	26.34	19.74
Percent non-Hispanic minority			156	4	37	12.76	6.27
Title 1	47	109	156				
**School Characteristics**							
School enrollment (2016–2017)			156	309	1231	626.07	183.50
Canyons School District	28	128	156				
Granite School District	62	94	156				
Jordan School District	34	122	156				
Murray School District	6	150	156				
Salt Lake City School District	26	130	156				

^a^ Chronic PM_2.5_ refers to the daily average of the modeled PM_2.5_ (i.e., particulate matter with diameters of 2.5 micrometers or smaller) concentrations (μg/m^3^) in each school’s tract for all days in 2016. ^b^ Frequency of peak PM_2.5_ is the number of days (in 2016) that the school tract’s PM_2.5_ concentration was over the 95th percentile of the daily mean of all tract days in the county (i.e., 22.80 μg/m^3^).

**Table 2:** Results of the generalized estimating equations (GEEs) predicting chronic (annual daily average, 2016) PM_2.5_ concentrations (Model 1) and the frequency of peak (number of days above 95th percentile, 2016) PM_2.5_ exposures (Model 2) at Salt Lake County public primary schools (*n* = 156).

Variable	Model 1		Model 2	
	Coefficient *p* (Standard Error)	95% Confidence Interval	Coefficient *p* (Standard Error)	95% Confidence Interval
Intercept	2.12 ***		17.37 ***	
District ^a^				
Canyons	−0.48 *** (0.04)	−0.56, −0.39	1.37 *** (0.30)	0.78, 0.96
Granite	−0.29 *** (0.05)	−0.37, −0.18	0.97 ** (0.34)	0.30, 1.64
Jordan	−0.72 *** (0.09)	−0.90, −0.55	1.46 *** (0.35)	0.77, 2.15
Murray	−0.12 *** (0.03)	−0.19, −0.06	3.09 *** (0.59)	1.94, 4.24
Total enrollment (standardized)	0.00 (0.02)	−0.03, 0.04	−0.10 (0.13)	−0.35, 0.16
School disadvantage	0.14 *** (0.02)	0.10, 0.17	1.28 *** (0.16)	0.98, 1.59

Note: Model 1 used an exchangeable working correlation matrix with an inverse Gaussian distribution and an identity link function. Model 2 used an unstructured working correlation matrix with a negative binomial distribution and an identity link function. ^a^ The reference category was Salt Lake City School District. *** *p* < 0.001, ** *p* < 0.01.

**Table 3:** Results of the Generalized Estimating Equations (GEEs) predicting the percentage of students below proficient in math at Salt Lake County public primary schools (*n* = 156).

Variable	Model 3		Model 4		Model 5		Model 6		Model 7	
	Coefficient *p* (Standard Error)	95% Confidence Interval	Coefficient *p* (Standard Error)	95% Confidence Interval	Coefficient *p* (Standard Error)	95% Confidence Interval	Coefficient *p* (Standard Error)	95% Confidence Interval	Coefficient *p* (Standard Error)	95% Confidence Interval
Intercept	−219.85 *** (46.01)	−310.02, −129.68	−50.59 *** (13.38)	−76.81, −24.37	−38.39 (32.41)	−101.91, 25.14	−9.32 (10.49)	−29.88, 11.25	8.86 (11.42)	13.24, 0.60
District ^a^										
Canyons	13.57 ** (5.47)	2.85, 24.29	−3.54 (3.23)	−9.86, 2.78	13.86 *** (3.42)	7.16, 20.56	8.95 *** (2.80)	3.47, 14.44	7.95 ** (2.60)	2.85, 13.05
Granite	25.91 *** (2.75)	20.51, 31.31	14.44 *** (2.51)	9.52, 19.34	26.14 *** (2.23)	21.77, 30.51	22.01 *** (2.37)	17.36, 26.67	20.51 *** (2.21)	16.18, 24.83
Jordan	31.72 *** (5.91	20.14, 43.31	8.74 * (3.82)	1.25, 16.22	27.39 *** (4.31)	18.94, 35.84	21.06 *** (3.34)	14.51, 27.06	19.93 *** (3.19)	13.68, 26.19
Murray	4.39 (3.32)	−2.12, 10.90	−10.44 * (4.24)	−18.74, −2.14	8.35 *** (2.16)	4.11, 12.58	3.25 (2.69)	−2.02, 8.53	1.31 (2.61)	−3.81, 6.43
Total enrollment (standardized)	−2.29 ** (0.87)	−3.99, −0.58	−2.44 ** (0.83)	−4.07, −0.82	−2.71 *** (0.85)	−4.38, −1.04	−2.65 *** (0.79)	−4.19, −1.11	−2.41 ** (0.71)	−3.80, −1.01
Daily annual average PM_2.5_	28.70 *** (5.43)	18.06, 39.33			6.49 (3.83)	−1.02, 14.00				
Frequency of peak PM_2.5_			4.09 *** (0.73)	2.65, 5.52			1.46 ** (0.58)	0.33, 2.60	0.65 (0.61)	−0.54, 1.83
School disadvantage					11.70 *** (1.15)	9.45, 13.94	10.73 *** (1.12)	8.54, 12.92	44.18 *** (12.24)	20.20, 68.17
DisadvantageXpeak PM_2.5_									−1.73 ** (0.64)	−2.98, −0.48

Note: The working correlation matrix for all models in this table was unstructured with a normal distribution and an identity link function. ^a^ The reference category was Salt Lake City School District. *** *p* < 0.001, ** *p* < 0.01, * *p* < 0.05.

**Table 4:** Results of the Generalized Estimating Equations (GEEs) predicting the percentage of students below proficient in English language arts (ELA) at Salt Lake County public primary schools (*n* = 156).

Variable	Model 8		Model 9		Model 10		Model 11		Model 12	
	Coefficient *p* (Standard Error)	95% Confidence Interval	Coefficient *p* (Standard Error)	95% Confidence Interval	Coefficient *p* (Standard Error)	95% Confidence Interval	Coefficient *p* (Standard Error)	95% Confidence Interval	Coefficient *p* (Standard Error)	95% Confidence Interval
Intercept	−210.09 *** (47.46)	−303.11, −117.07	−46.27 ** (16.86)	−79.31, −13.23	−29.55 (46.32)	−120.33, 61.23	−13.21 (13.25)	−39.17, 12.76	−3.79 (16.75)	−36.63, 29.04
District ^a^										
Canyons	7.37 (5.67)	−3.74, 18.47	−8.31 ** (3.16)	−14.51, −2.11	8.40 ** (3.86)	0.84, 16.00	3.70 (2.66)	−1.51, 8.90	2.55 (2.78)	−2.90, 8.00
Granite	23.54 *** (3.47)	16.73, 30.35	13.04 *** (2.99)	7.19, 18.90	23.45 ***(3.07)	17.43, 29.46	19.83 *** (2.76)	14.42, 25.25	18.25 *** (2.93)	12.50, 24.00
Jordan	24.55 *** (6.21)	12.38, 36.71	4.40 (3.69)	−2.85, 11.64	20.69 *** (4.68)	11.51, 29.87	14.75 *** (2.95)	9.00, 20.53	13.60 *** (3.23)	7.27, 19.94
Murray	2.32 (3.89)	−5.29, 9.94	−12.19 ** (4.36)	−20.73, −3.65	6.32 ** (2.58)	1.28, 11.37	0.34 (3.15)	−5.83, 6.52	−1.80 (3.53)	−8.71, 5.12
Total enrollment	−2.01 ** (0.93)	−3.83, −0.19	−3.36 ** (1.14)	−5.60, −1.12	−2.47 ** (0.91)	−4.24, −0.70	−2.63 *** (0.81)	−4.22, −1.05	−2.62 ** (0.88)	−4.40, −0.90
Daily annual average PM_2.5_	27.81 *** (5.58)	16.87, 38.74			5.68 (5.48)	−5.06, 16.42				
Frequency of peak PM_2.5_			3.92 *** (0.92)	2.12, 5.73			1.79 ** (0.74)	0.35, 3.23	1.41 (0.88)	−0.32, 3.14
Disadvantage					11.53 *** (1.39)	8.18, 14.25	10.00 *** (1.42)	7.30, 12.80	33.99 ** (16.97)	0.73, 67.25
DisadvantageXpeak PM_2.5_									−1.27 (0.88)	−2.98, 0.45

Note: The working correlation matrix for all models in this table was unstructured with a normal distribution and an identity link function. ^a^ The reference category was Salt Lake City School District. *** *p* < 0.001, ** *p* < 0.01.
